# Research on trajectory tracking control of tracked vehicles based on hydraulic motor system identification and Laguerre-MPC

**DOI:** 10.1371/journal.pone.0346824

**Published:** 2026-06-26

**Authors:** Sheng Jin, Daqing Zhang, Qijun Tang, Haoyu Huang, Hongjie Luo, Yuming Zhao, Kang Wu

**Affiliations:** 1 College of Mechanical and Electrical Engineering, Hunan Agricultural University, Changsha, Hunan, China; 2 Sunward Intelligent Equipment Co., Ltd., Changsha, Hunan, China; Southwest Jiaotong University, CHINA

## Abstract

To address the high computational cost of model predictive control and the high complexity associated with physics-based modeling of hydraulic drive systems in trajectory tracking of tracked unmanned vehicles, a hierarchical control framework is proposed to enhance trajectory tracking performance. In the upper-layer control, a Laguerre function-based model predictive control (Laguerre-MPC) strategy is developed to reduce the computational burden while maintaining control performance. In the lower-layer control, Hammerstein-Wiener identification is employed to establish control models for the left and right hydraulic motors, thereby avoiding the modeling complexity inherent to hydraulic systems. Moreover, a proportional-integral-derivative (PID) controller is incorporated into the lower-layer control to improve disturbance rejection during operation. Simulation results indicate that Laguerre-MPC yields substantially lower computational complexity than conventional MPC, with the average time required for a single optimization being only 24.3% of that required by conventional MPC, which improves the real-time capability of the control algorithm. Furthermore, field experiments are conducted on a tracked unmanned vehicle equipped with relevant sensors, including speed tracking and trajectory tracking tests under specified operating conditions. The results confirm the effectiveness of the proposed framework: compared with the conventional MPC + PID scheme, the proposed method achieves higher tracking accuracy, improving the average speed tracking accuracy by 28.7%, the straight-line trajectory tracking accuracy (root mean square error) by 65.5%, and the curved trajectory tracking accuracy (root mean square error) by 10.1%. The proposed framework provides a practical and efficient solution for trajectory tracking control of tracked unmanned vehicles with clear engineering applicability.

## Introduction

Tracked vehicles are widely deployed in unstructured environments, including disaster rescue and agricultural operations, because of high mobility and strong impact resistance. These platforms perform mission-critical tasks such as emergency response and material transport [1,2]. Precise trajectory tracking, however, is difficult to achieve because hydraulic systems exhibit pronounced nonlinearity and time delay [3]. Track slippage on unstructured terrains can further degrade tracking accuracy, but this factor is outside the scope of the present study. Therefore, enhancing trajectory tracking accuracy for hydraulically driven tracked unmanned platforms is a key enabling technology for intelligent operation [4].

Trajectory tracking and control for autonomous vehicles have been widely investigated in both domestic and international studies. Representative approaches include PID control [5], preview control [6], linear quadratic regulator [7], sliding mode control [8], fuzzy control [9], and adaptive control [10]. However, when applied to nonlinear systems, many of these methods show reduced effectiveness and provide limited capability for addressing constraint-related problems.

In contrast, model predictive control (MPC) provides clear advantages for tracked unmanned platforms by explicitly handling constraints and enabling optimal control, and it has been widely used in autonomous trajectory tracking [11,12]. Li et al [13] integrated the particle swarm optimization algorithm into MPC to optimize the weight matrices online, thereby improving trajectory tracking accuracy and dynamic response.Wang et al [14] adjusted MPC weights using a fuzzy adaptive weight control algorithm, which improved vehicle path tracking accuracy. You et al [15] combined MPC with an adaptive neuro-fuzzy inference system to develop a control strategy for ground mobile robots, and validated its effectiveness through real-vehicle experiments. Guo et al [16] proposed an LNMPC + improved iLQR strategy that generates a unified yaw rate reference based on front-wheel steering angle feedback, thereby enhancing handling stability, robustness, and real-time performance for distributed drive electric vehicles. Botes et al [17] incorporated road friction into a nonlinear predictive model to estimate dynamic constraints, including slip angles, yaw rates, and rollover risks, thereby improving lateral stability. Nevertheless, most of these methods have been validated only in simulation, while practical effects induced by inherent dynamics, nonlinearities, and response delays in hydraulic and transmission systems are often not fully considered. In addition, overly long control horizons impose a substantial computational burden.

In hydraulic system control, sliding mode control [18] and active disturbance rejection control [19] can reduce reliance on accurate models during operation. Nevertheless, hydraulic mechanisms exhibit inherent nonlinearity, friction, and leakage, which increase model uncertainty and degrade model fidelity. Consequently, system identification-based approaches have attracted growing attention in recent years. These approaches construct mathematical models from input-output data, thereby alleviating the adverse effects associated with model uncertainty [20].

Hulttinen et al [21] investigated a hydraulic manipulator and identified key parameters, including friction and flow coefficients, using parameter identification methods. Subsequent motion tests showed a substantial improvement in tracking performance. Chen et al [22] integrated theoretical modeling with system identification to develop a hydrostatic drive model, which improved motion control of tracked vehicles. Zhang et al [23] studied an electro-hydrostatic servo system and applied least squares and genetic algorithms to identify critical parameters, thereby increasing model accuracy. Xu et al [24] combined theoretical modeling and system identification to derive the transfer function of the control model for an asymmetric hydraulic cylinder servo pump system, providing practical guidance for hydraulic control applications. However, despite these approaches, obtaining a simplified hydraulic system model remains difficult, and parameter identification is often limited by high time consumption and high cost.

In summary, two key challenges remain in existing studies. First, traditional MPC involves substantial computational complexity, which makes real-time implementation difficult to guarantee. Second, hydraulic drive systems are strongly nonlinear and difficult to model accurately, and modeling accuracy is often achieved at the expense of increased complexity. As a result, trajectory tracking for tracked vehicles rarely satisfies real-time and high-precision requirements simultaneously. To overcome these limitations, a hierarchical control framework is proposed. In the upper layer, Laguerre functions are introduced to reduce the decision-variable dimension of conventional MPC, thereby markedly decreasing the online computational burden. In the lower layer, a hydraulic motor model is identified using the Hammerstein-Wiener method, and a PID controller is incorporated for speed correction and disturbance compensation. The resulting architecture improves the real-time feasibility of MPC while enhancing actuator dynamic response, enabling coordinated improvements in tracking accuracy and computational complexity. Moreover, the proposed framework is transferable to real-time optimal control problems in complex systems, such as chemical processes, power systems, aerospace, metallurgical equipment, marine engineering, and new energy applications. The main contributions are summarized as follows:

(1) An upper-layer Laguerre-based model predictive control (Laguerre-MPC) strategy is developed. By employing Laguerre orthogonal basis functions for dimensionality-reduced optimization of MPC control variables, the computational load is significantly reduced without sacrificing control precision, thereby supporting real-time trajectory tracking. This layer provides accurate control commands to the lower-layer hydraulic actuators and establishes the basis for high-precision tracking performance.
(2) In the lower layer, a nonlinear mapping between hydraulic motor input (control current) and output (rotational speed) is established via data acquisition, which avoids the cumbersome physical modeling procedure. A proportional-integral-derivative (PID) controller is integrated to perform real-time speed regulation and disturbance compensation. Owing to its fast response and straightforward tuning, the PID controller dynamically corrects the speed commands in real time, compensating for the limited anti-interference capability of a stand-alone identification model and ensuring accurate tracking of the upper-layer Laguerre-MPC commands.
(3) Comprehensive experiments, including speed tracking, straight-line trajectory tracking, and curved trajectory tracking, are conducted on a hydraulically driven tracked unmanned vehicle. The results demonstrate the effectiveness and superiority of the proposed control strategy.

The remainder of this paper is organized as follows. The Tracked Vehicle Model section presents a hydraulically driven tracked unmanned vehicle model, including the tracked-vehicle kinematic model and an identification method for the hydrostatic drive system model. Next, the Controller Design section details the hierarchical control strategy and its implementation. The Experiment section then reports trajectory-tracking tests conducted on the fully hydraulic tracked unmanned vehicle, with results for speed tracking, straight-line trajectory tracking, and curved trajectory tracking. Finally, the Conclusions and Discussion section summarizes the main findings and outlines directions for future work.

## Tracked vehicle model

### Kinematic model of tracked vehicle

**Fig 1:**
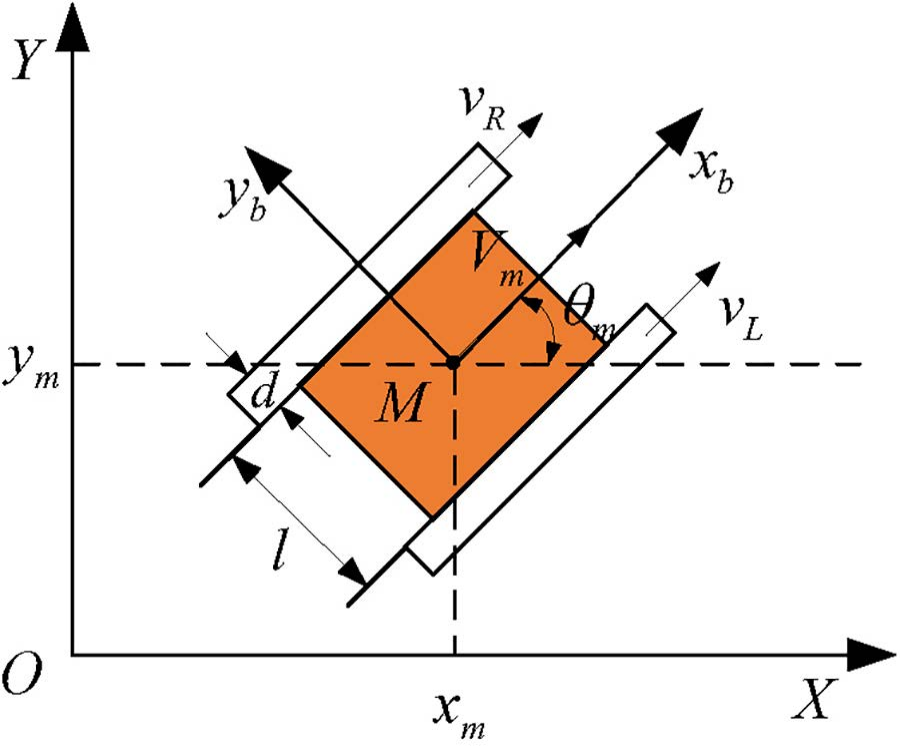
Schematic diagram of tracked vehicle motion.

Initially, a global coordinate system *XOY* and a local coordinate system $x_{b}My_{b}$ are defined. The local coordinate system is centered at the centroid *M* of the unmanned vehicle. Here, $v_{R}$ and $v_{L}$ denote the velocities of the right and left tracks, respectively. *L* represents the distance between the track centers, *d* indicates the track width, and θ signifies the orientation angle. A schematic diagram of the kinematic model is illustrated in Fig 1, and the corresponding kinematic equations [25] are presented in Eq (1):


$$\begin{array}{c} \begin{array}{c} {\dot{x}}_{c}=\left[\begin{array}{c} \dot{x}\\ \dot{y}\\ \dot{\theta } \end{array}\right]=\left[\begin{array}{cc} \cos\theta & 0\\ \sin\theta & 0\\ 0 & 1 \end{array}\right]u\; \end{array} \end{array}$$ (1)

where: $u={\left[\begin{array}{cc} v & \omega \end{array}\right]}^{T}$

By performing a Taylor expansion of Eq (1) around the reference trajectory pose ${\left[\begin{array}{ccc} x_{r} & y_{r} & \theta_{r} \end{array}\right]}^{T}$ and reference velocity ${\left[\begin{array}{cc} v_{r} & \omega_{r} \end{array}\right]}^{T}$, the following is obtained:


$$\begin{array}{c} \left\{ \begin{array}{c} \dot{\tilde{x}}=A_{c}{\tilde{x}}_{c}+B_{c}\tilde{u}\\ y_{c}=C_{c}{\tilde{x}}_{c} \end{array}\right. \end{array}$$ (2)

where:


$$\begin{array}{c} {\tilde{x}}_{c}=[\begin{array}{c} x-x_{r}\\ y-y_{r}\\ \theta -\theta_{r} \end{array}],\;\tilde{u}=[\begin{array}{c} v-v_{r}\\ \omega -\omega_{r} \end{array}],\;A_{c}=[\begin{array}{ccc} 0 & 0 & -v_{r}\sin\theta_{r}\\ 0 & 0 & v_{r}\cos\theta_{r}\\ 0 & 0 & 0 \end{array}],\;B_{c}=[\begin{array}{cc} \cos\theta_{r} & 0\\ \sin\theta_{r} & 0\\ 0 & 1 \end{array}],\;C_{c}=I \end{array}$$

Discretizing Eq (2) yields:


$$\begin{array}{c} \left\{ \begin{array}{c} \tilde{x}(k+1)=A_{m}\tilde{x}(k)+B_{m}\tilde{u}(k)\\ \tilde{y}(k)=C_{m}\tilde{x}(k) \end{array}\right. \end{array}$$ (3)

where: $A_{m}=I+T\cdot A_{c}$, $B_{m}=T\cdot B_{c}$, $C_{m}=I$.

Applying the difference operation to Eq (3), defining $\Delta \tilde{x}(k)=\tilde{x}(k+1)-\tilde{x}(k)$, Δu(k)=u(k+1)−u(k) and setting $x(k)={\left[\Delta \tilde{x}(k)\;\tilde{y}(k)\right]}^{T}$, the state-space equation is derived as shown in Eq (4).


$$\begin{array}{c} \left\{ \begin{array}{c} x(k+1)=Ax(k)+B\Delta u(k)\\ \tilde{y}(k)=Cx(k) \end{array}\right. \end{array}$$ (4)

where: $A=[\begin{array}{cc} A_{m} & O_{m}^{T}\\ C_{m}A_{m} & I \end{array}]$, $B=[\begin{array}{c} B_{m}\\ C_{m}B_{m} \end{array}]$, $C=[\begin{array}{cc} O_{m} & I \end{array}]$

With the control horizon defined as $N_{c}$ and the prediction horizon as $N_{p}$, the state prediction equation at any time instant $k_{i}$ is expressed as:


$$\begin{array}{c} Y=Fx(k_{i})+\Phi \Delta U \end{array}$$ (5)

where:


$$\begin{array}{c} Y=[\begin{array}{c} y(k_{i}+1|k_{i})\\ y(k_{i}+2|k_{i})\\ \vdots \\ y(k_{i}+N_{p}|k_{i}) \end{array}],\;F=[\begin{array}{c} CA\\ CA^{2}\\ \vdots \\ CA^{N_{p}} \end{array}],\;x(k_{i})=[\begin{array}{c} x(k_{i}+1|k_{i})\\ x(k_{i}+2|k_{i})\\ \vdots \\ x(k_{i}+N_{p}|k_{i}) \end{array}] \end{array}$$

$$\begin{array}{c} \Phi =[\begin{array}{cccc} CB & 0 & \cdots & 0\\ CAB & CB & \cdots & 0\\ \vdots & \vdots & \ddots & \vdots \\ CA^{N_{p}-1}B & CA^{N_{p}-2}B & \cdots & CA^{N_{p}-N_{c}}B \end{array}],\;\Delta U=[\begin{array}{c} \Delta u(k_{i})\\ \Delta u(k_{i}+1)\\ \vdots \\ \Delta u(k_{i}+N_{c}-1) \end{array}] \end{array}$$

### System identification of the hydrostatic drive system

**Fig 2:**
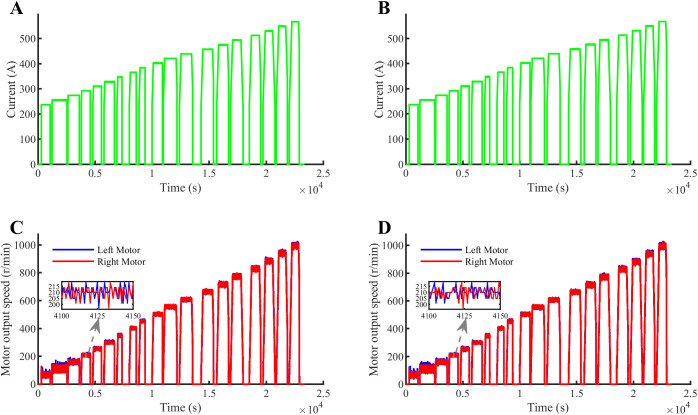
Identification test results: **(A)** System identification input data; **(B)** System identification verification input data; **(C)** System identification output data; **(D)** System identification output data.

A system identification model is developed using the solenoid valve input current and the hydraulic motor output rotational speed [22]. In the identification experiment, a stepwise current excitation signal is applied, and its amplitude is strictly limited to the non-saturated operating range of the pump-motor. With a constant step size of 0.018 A, the current increases linearly from 0.237 A to 0.567 A. This interval matches the effective adjustment range (225 mA–600 mA) of the solenoid valve for the travel motor in the tracked hydrostatic system, thereby covering the key nonlinear region from zero current to the start threshold current while maintaining bench-test operability and safety. Two sets of input-output data were obtained from bench tests, as shown in Fig 2; one set is used for system identification, and the other is used for model simulation validation. The controller sampling period is set to 0.05 s.

**Fig 3:**
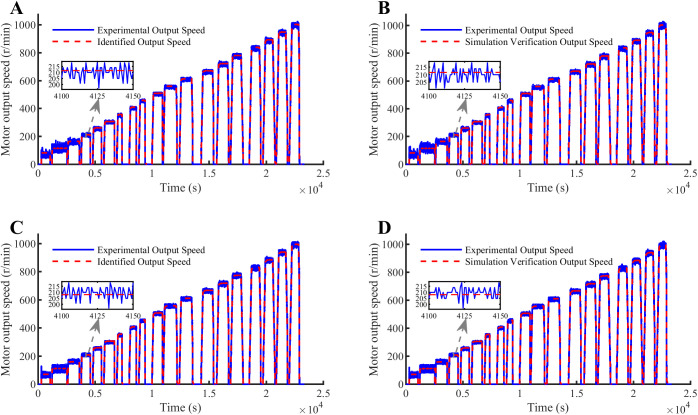
System identification results and simulation verification: **(A)** Left motor identification result; **(B)** Left motor verification result; **(C)** Right motor identification result; **(D)** Right motor verification result.

Given the inherent differences between the left and right hydraulic drive systems, separate identification and simulation validation were conducted for each side. As illustrated in the experimental results in Fig 2 and the current–speed relationship validation for the left and right motors in Fig 3, the Hammerstein-Wiener model exhibited remarkable tracking performance, achieving a fitting accuracy of 90% for both motor models. The Hammerstein-Wiener model comprises three core components: an input nonlinear block, *v*(*t*) = *f*(*u*(*t*)); a linear dynamic system, *w*(*t*) = *G*(*q*)*v*(*t*); and an output nonlinear block, *y*(*t*) = *h*(*w*(*t*)).

## Controller design

### Overall framework

**Fig 4:**
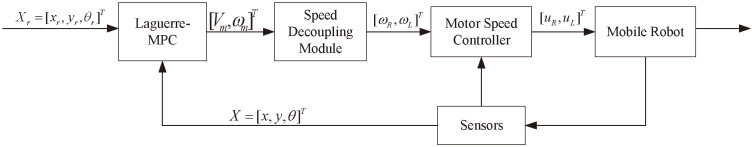
Overall structure of the tracked unmanned vehicle controller.

The overall architecture of the vehicle controller is illustrated in Fig 4. In the upper layer, Laguerre-MPC functions as the position-loop controller to track the desired trajectory $X_{r}=[x_{r},y_{r},\theta_{r}{]}^{T}$. Laguerre-MPC outputs the commanded linear velocity and angular velocity $[V_{m},\omega_{m}{]}^{T}$ of the unmanned vehicle. These commands are mapped by a velocity decoupling module into the rotational speeds of the left and right motors $[\omega_{R},\omega_{L}{]}^{T}$, which are used as reference inputs for the motor controllers. Using motor speed error signals, the motor speed controllers regulate pump displacement by modulating the input current, thereby driving the actual motor speeds to the commanded values and achieving the control objective. In addition, fused RTK-GPS and track-speed odometer measurements provide feedback of the robot’s pose $X=[x,y,\theta {]}^{T}$ and angular velocities $[\omega_{1,r},\omega_{1,l}{]}^{T}$, which are delivered to the controller for closed-loop operation.

Within the velocity decoupling module, the vehicle’s linear velocity and angular velocities are transformed into the rotational speeds of the left and right motors as follows:


$$\begin{array}{c} \left[\begin{array}{c} v_{m}\\ \omega_{m} \end{array}]=[\begin{array}{cc} \frac{1}{2} & \frac{1}{2}\\ \frac{2}{l+d} & -\frac{2}{l+d} \end{array}][\begin{array}{c} v_{R}\\ v_{L} \end{array}]=[\begin{array}{cc} \frac{b}{2} & \frac{b}{2}\\ \frac{2b}{l+d} & -\frac{2b}{l+d} \end{array}][\begin{array}{c} \omega_{R}\\ \omega_{L} \end{array}\right] \end{array}$$ (6)

where $v_{m}$ represents the linear velocity of the vehicle, $\omega_{m}$ represents the angular velocity of the vehicle, and $\omega_{L}$, $\omega_{R}$ indicate the rotational speeds of the left and right hydraulic motors, respectively.

### Design of the MPC control algorithm based on laguerre functions

**Fig 5:**
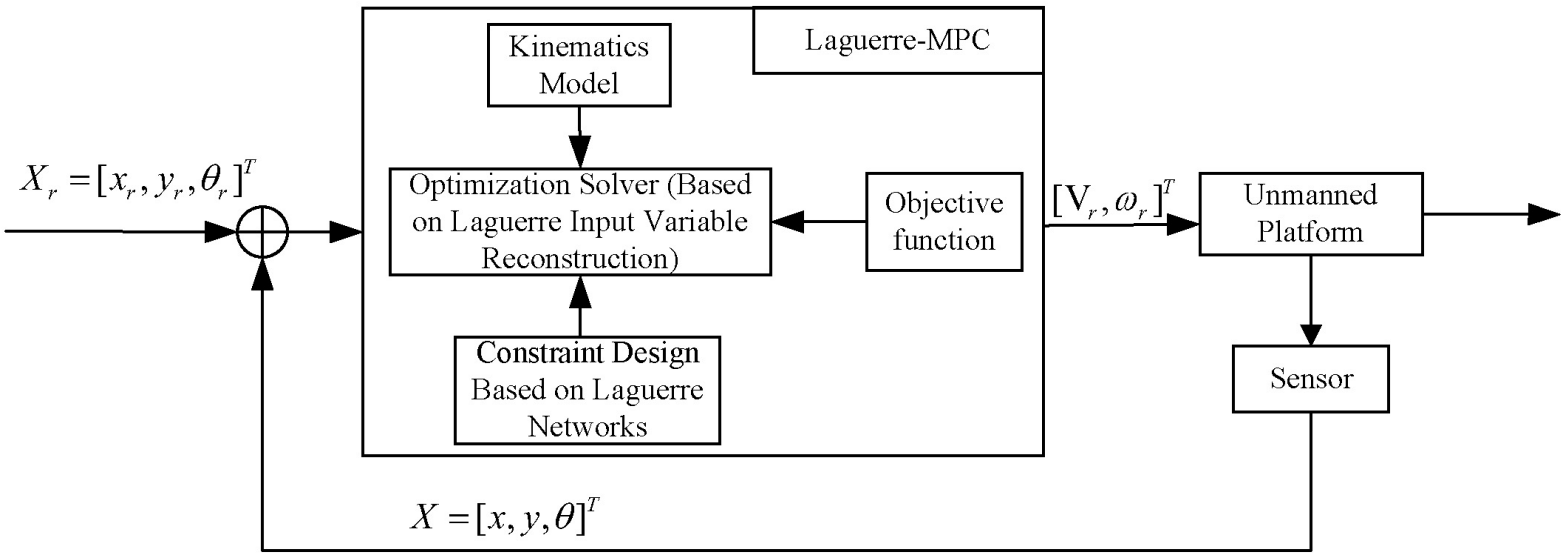
Structure diagram of Laguerre-MPC control.

This section presents an MPC formulation based on Laguerre functions, and the corresponding control algorithm is illustrated in Fig 5. The algorithm design incorporates the kinematic model and system constraints, while Laguerre functions are embedded in the optimization procedure. To reduce the computational burden, the control sequence ΔU is approximated using Laguerre functions; this approach retains the control benefits associated with a long prediction horizon and maintains manageable computational complexity [26]. The Laguerre functions are defined as follows [27].


$$\begin{array}{c} \Gamma_{1}(z)=\frac{\sqrt{1-a^{2}}}{1-az^{-1}} \end{array}$$ (7)

$$\begin{array}{c} \Gamma_{2}(z)=\frac{\sqrt{1-a^{2}}}{1-az^{-1}}\left(\frac{z^{-1}-a}{1-az^{-1}}\right) \end{array}$$ (8)

$$\begin{array}{c} \vdots \end{array}$$

$$\begin{array}{c} \Gamma_{N}(z)=\frac{\sqrt{1-a^{2}}}{1-az^{-1}}{\left(\frac{z^{-1}-a}{1-az^{-1}}\right)}^{N-1} \end{array}$$ (9)

Where, *a* represents the pole, with a value constrained to the range 0≤a<1; N denotes the order of the Laguerre function. After performing the inverse z-transform on each term in Eqs (7) to (9), the discrete-time Laguerre function can be expressed in vector form as:


$$\begin{array}{c} L(k)={\left[\begin{array}{cccc} l_{1}(k) & l_{2}(k) & \ldots & l_{N}(k) \end{array}\right]}^{T} \end{array}$$ (10)

where $l_{i}(k)$ corresponds to the Z-transform of $\Gamma_{i}(z,a)$, denoted as $\Gamma_{N}(z)=\Gamma_{k-1}(z)\frac{z^{-1}-a}{1-az^{-1}}$. Eq (10) can be reformulated as:


$$\begin{array}{c} L(k+1)=A_{l}L(k) \end{array}$$ (11)

where: $\beta =1-a^{2}$, and $L(0{)}^{T}=\sqrt{\beta }[\begin{array}{c} 1,-a,a^{2},-a^{3},\ldots ,(-1{)}^{N-1}a^{N-1} \end{array}]$


$$\begin{array}{c} A_{l}=[\begin{array}{cccccc} \alpha & 0 & 0 & 0 & \ldots & 0\\ \beta & \alpha & 0 & 0 & \ldots & 0\\ -\alpha \beta & \beta & \alpha & 0 & \ldots & 0\\ \alpha^{2}\beta & -\alpha \beta & \beta & \alpha & \ldots & 0\\ \vdots & \vdots & \vdots & \vdots & \ddots & \vdots \\ (-1{)}^{N-2}\alpha^{N-2}\beta & (-1{)}^{N-3}\alpha^{N-3}\beta & (-1{)}^{N-4}\alpha^{N-4}\beta & (-1{)}^{N-5}\alpha^{N-5}\beta & \ldots & \alpha \end{array}] \end{array}$$

Assuming the impulse response of a stable system at time *k* is *H*(*k*), and for a specified parameter *N*, the discrete-time impulse response of the dynamic system can be approximated using the Laguerre model as:


$$\begin{array}{c} H(k)=c_{1}l_{1}(k)+c_{2}l_{2}(k)+\ldots +c_{N}l_{N}(k) \end{array}$$ (12)

The time series of the control increment Δu is approximated by a linear combination of Laguerre basis functions. Thus, at any instant *ki*, it can be expressed as:


$$\begin{array}{c} \Delta u(k_{i}+k)=\sum_{j=1}^{\infty }c_{j}(k_{i})l_{j}(k)=L(k{)}^{T}\eta \end{array}$$ (13)

where: $\eta ={\left[\begin{array}{cccc} c_{1} & c_{2} & \ldots & c_{N} \end{array}\right]}^{T}$.

By combining Eqs (4), (5) and (13), the state at time *m* relative to time $k_{i}$ can be derived as:


$$\begin{array}{c} x(k_{i}+m|k_{i})=A^{m}x(k_{i})+\sum_{i=0}^{m-1}A^{m-i-1}BL(i{)}^{T}\eta \end{array}$$ (14)

$$\begin{array}{c} y(k_{i}+m|k_{i})=CA^{m}x(k_{i})+\sum_{i=0}^{m-1}CA^{m-i-1}BL(i{)}^{T}\eta \end{array}$$ (15)

The cost function and associated constraint in the MPC optimization problem are formulated as:


$$\begin{array}{c} J=\sum_{m=1}^{N_{p}}(x(k_{i}+m|k_{i}){)}^{T}Q\;x(k_{i}+m|k_{i})+\Delta U^{T}R_{L}\Delta U \end{array}$$ (16)

$$\begin{array}{c} \text{s.t.}\;u_{min}\leq u(k_{i}+m)\leq u_{max} \end{array}$$ (17a)

$$\begin{array}{c} \Delta u_{min}\leq \Delta u(k_{i}+m)\leq \Delta u_{max} \end{array}$$ (17b)

$$\begin{array}{c} x_{min}\leq x(k_{i}+m|k_{i})\leq x_{max} \end{array}$$ (17c)

where: $\Delta u(k_{i}+m)=[\begin{array}{cccc} l_{1}(m) & l_{2}(m) & \ldots & l_{N}(m) \end{array}]\eta$.

Leveraging the orthogonal properties of the Laguerre functions, the following relationship can be derived:


$$\begin{array}{c} \Delta U^{T}\bar{R}\Delta U=\sum_{m=0}^{N_{p}}\Delta u(k_{i}+m{)}^{T}r_{w}\;\Delta u(k_{i}+m)=\eta^{T}R_{L}\eta \end{array}$$ (18)

By integrating Eqs (14), (16), and (18), the cost function becomes:


$$\begin{array}{c} J=\eta^{T}\Omega \eta +2\eta^{T}\Psi x(k_{i})+\sum_{m=1}^{N_{p}}x(k_{i}{)}^{T}(A^{T}{)}^{m}QA^{m}x(k_{i}) \end{array}$$ (19)

As evident from Eq (19), the cost function depends on η, while the third $\sum_{m=1}^{N_{p}}x(k_{i}{)}^{T}(A^{T}{)}^{m}QA^{m}x(k_{i})$ is independent of it. Consequently, the cost function can be rewritten as shown in Eq (20):


$$\begin{array}{c} J=\eta^{T}\Omega \eta +2\eta^{T}\Psi x(k_{i}) \end{array}$$ (20)

where:


$$\begin{array}{c} \Omega =\sum_{m=1}^{N_{p}}\varphi (m)Q\varphi (m{)}^{T}+R_{L},\;\Psi =\sum_{m=1}^{N_{p}}\varphi (m)QA^{m},\;\varphi (m{)}^{T}=\sum_{i=0}^{m-1}A^{m-i-1}BL(i{)}^{T} \end{array}$$

By reformulating the constraints and substituting Eq (13) into (17), the updated constraints are obtained as:


$$\begin{array}{c} u_{\text{min}}\leq L(k{)}^{T}\eta +u(k_{i}-1)\leq u_{\text{max}} \end{array}$$ (21)

$$\begin{array}{c} \Delta u_{\text{min}}\leq L(k{)}^{T}\eta \leq \Delta u_{\text{max}} \end{array}$$ (22)

$$\begin{array}{c} x_{\text{min}}\leq \hat{x}(k_{i}+m|k_{i})\leq x_{\text{max}} \end{array}$$ (23)

Thus, the constraint on the control increment can be expressed as:


$$\begin{array}{c} \left\{ \begin{array}{c} M_{0}\eta \leq \Delta U_{max}\\ -M_{0}\eta \leq -\Delta U_{min} \end{array}\right. \end{array}$$ (24)

Similarly, the constraint on the control input can be reformulated as:


$$\begin{array}{c} \left\{ \;\;\begin{array}{c} M_{1}\eta \leq U_{max}-\overset{―}{u}(k_{i}-1)\\ -M_{1}\eta \leq -U_{min}+\overset{―}{u}(k_{i}-1) \end{array}\right. \end{array}$$ (25)

where:


$$\begin{array}{c} M_{0}=[\begin{array}{cccc} L_{1}(m{)}^{T} & o_{1}^{T} & \ldots & o_{1}^{T}\\ o_{1}^{T} & L_{2}(m{)}^{T} & \ldots & o_{1}^{T}\\ \vdots & \vdots & \ddots & \vdots \\ o_{1}^{T} & o_{1}^{T} & \ldots & L_{m}(m{)}^{T} \end{array}],\;M_{1}=[\begin{array}{cccc} \sum_{i=0}^{k-1}L_{1}(i{)}^{T} & o_{1}^{T} & \ldots & o_{1}^{T}\\ o_{1}^{T} & \sum_{i=0}^{k-1}L_{2}(i{)}^{T} & \ldots & o_{1}^{T}\\ \vdots & \vdots & \ddots & \vdots \\ o_{1}^{T} & o_{1}^{T} & \ldots & \sum_{i=0}^{k-1}L_{m}(i{)}^{T} \end{array}] \end{array}$$

Using Eq (3), the predicted value at time $k_{i}$ is derived as:


$$\begin{array}{c} X(k_{i})=A_{c}\hat{x}(k_{i})+B_{c}U(k_{i}) \end{array}$$ (26)

where:


$$\begin{array}{c} X(k_{i}|k_{i})=[\begin{array}{c} \hat{x}(k_{i}+1|k_{i})\\ \hat{x}(k_{i}+2|k_{i})\\ \vdots \\ \hat{x}(k_{i}+N_{p}|k_{i}) \end{array}],\;U(k_{i})=[\begin{array}{c} \hat{u}(k_{i})\\ \hat{u}(k_{i}+1)\\ \vdots \\ \hat{u}(k_{i}+N_{c}-1) \end{array}],\;A_{e}=[\begin{array}{c} A\\ A^{2}\\ \vdots \\ A^{N_{p}} \end{array}],\;B_{c}=[\begin{array}{cccc} B & 0 & \ldots & 0\\ AB & B & \vdots & 0\\ \vdots & \vdots & \ddots & \vdots \\ A^{N_{c}-1}B & A^{N_{c}-2}B & \ldots & B \end{array}] \end{array}$$

Substituting Eqs (26) into (23) yields:


$$\begin{array}{c} x_{min}\leq A_{c}\hat{x}(k)+B_{c}U(k_{i})\leq x_{max} \end{array}$$ (27)

The constraint on the state variables can be rewritten as:


$$\begin{array}{c} \left\{ \begin{array}{c} B_{c}M_{1}\eta \leq x_{max}-A_{c}\hat{x}(k)-B_{c}\hat{u}(k_{i}-1)\\ -B_{c}M_{1}\eta \leq -x_{min}+A_{c}\hat{x}(k)+B_{c}\hat{u}(k_{i}-1) \end{array}\right. \end{array}$$ (28)

After consolidating, the optimization problem is expressed as:


$$\begin{array}{c} J=\frac{1}{2}\eta^{T}\Omega \eta +\eta^{T}\Psi x(k_{i}) \end{array}$$ (29)

$$\begin{array}{c} \text{s.t.}\;M\eta \leq \gamma \end{array}$$ (30)

where:


$$\begin{array}{c} \Omega =\sum_{m=1}^{Np}\varphi (m)Q\varphi (m{)}^{T}+R_{L},\;\Psi =\sum_{m=1}^{Np}\varphi (m)QA^{m},\;[\begin{array}{c} M_{0}\\ -M_{0}\\ M_{1}\\ -M_{1}\\ B_{c}M_{1}\\ -B_{c}M_{1} \end{array}]\eta \leq [\begin{array}{c} \Delta U\max\\ -\Delta U\min\\ U_{\max}-\bar{u}(k_{i}-1)\\ -U_{\min}+\bar{u}(k_{i}-1)\\ x_{\max}-A_{c}\tilde{x}(k)-B_{c}\bar{u}(k_{i}-1)\\ -x_{\min}+A_{c}\tilde{x}(k)+B_{c}\bar{u}(k_{i}-1) \end{array}] \end{array}$$

Where η being the variable to be optimized. $\Omega \in {\mathbb{R}}^{n\times n}$ represents a symmetric positive definite matrix, $\Psi \in {\mathbb{R}}^{n}$ denotes the coefficient vector of the linear term, $M\in {\mathbb{R}}^{m\times n}$ signifies the constraint matrix, and $\gamma \in {\mathbb{R}}^{m}$ indicates the offset vector of the constraints.

For the constrained cost function described above, the optimal solution can be iteratively computed using the Lagrange multiplier method and Hildreth’s QP approach.

### Stability analysis

Assumption:A terminal constraint is added to the receding horizon optimization as $x(k+N_{P}|k)=0$, where $x(k+N_{P}|k)$ denotes the terminal state generated by the control sequence $\Delta u(k+m)=L(m{)}^{T}\eta$ ($m=0,1,\ldots ,N_{P}$).

For each sampling time *k*, there exists a parameter vector $\eta^{k}$ such that the cost function *J* attains its minimum value subject to inequality constraints and the terminal equality constraint $x(k+N_{P}|k)=0$.

Definition of Lyapunov Function:


$$\begin{array}{c} V(x(k),k)=\sum_{m=1}^{N_{P}}x(k+m|k{)}^{T}Qx(k+m|k)+\sum_{m=0}^{N_{P}-1}\Delta u(k+m{)}^{T}R\Delta u(k+m)\\ \text{s.t.}\;x(k+N_{P}|k)=0 \end{array}$$ (31)

At time *k* + 1, the Lyapunov function becomes:


$$\begin{array}{c} V(x(k+1),k+1)=\sum_{m=1}^{N_{P}}x(k+1+m|k+1{)}^{T}Qx(k+1+m|k+1)+\sum_{m=0}^{N_{P}-1}\Delta u(k+1+m{)}^{T}R\Delta u(k+1+m) \end{array}$$ (32)

The one-step state recursion is given by x(k+1)=Ax(k)+BΔu(k). By shifting the entire control sequence at time *k*, i.e., $\left\{L(0{)}^{T}\eta^{k},L(1{)}^{T}\eta^{k},\ldots ,L(N_{P}-1{)}^{T}\eta^{k}\right\}$, forward by one step and appending a zero at the end of the sequence, a feasible control sequence at time *k* + 1 is obtained as:


$$\begin{array}{c} \left\{L(0{)}^{T}\eta^{k},L(1{)}^{T}\eta^{k},\ldots ,L(N_{P}-1{)}^{T}\eta^{k},0\right\} \end{array}$$ (33)

Denote the cost corresponding to this feasible sequence as $\hat{V}(x(k),k+1)$. Since the cost of the optimal solution is necessarily no greater than that of any feasible solution, we have:


$$\begin{array}{c} V(x(k+1),k+1)\leq \hat{V}(x(k),k+1) \end{array}$$ (34)

Combining with the terminal constraint $x(k+N_{P}|k)=0$, the difference of the Lyapunov function is derived as follows:


$$\begin{array}{c} \hat{V}(x(k+1),k+1)-V(x(k),k)=-x(k+1{)}^{T}Qx(k+1)-\Delta u(k{)}^{T}R\Delta u(k) \end{array}$$ (35)

It then follows that:


$$\begin{array}{c} V(x(k+1),k+1)-V(x(k),k)\leq -x(k+1{)}^{T}Qx(k+1)-\Delta u(k{)}^{T}R\Delta u(k) \end{array}$$ (36)

Since both *Q* and *R* are positive definite matrices, the right-hand side is negative semi-definite, and they are simultaneously zero if and only if *x*(*k* + 1) = 0 and Δu(k)=0. This indicates that the Lyapunov function V(x(k),k) is strictly decreasing with the sampling time. According to the Lyapunov asymptotic stability theorem, a Lyapunov function that satisfies the conditions of positive definiteness and strict decrease can guarantee the asymptotic stability of the closed-loop system.

### Motor speed control module based on system identification

**Fig 6:**
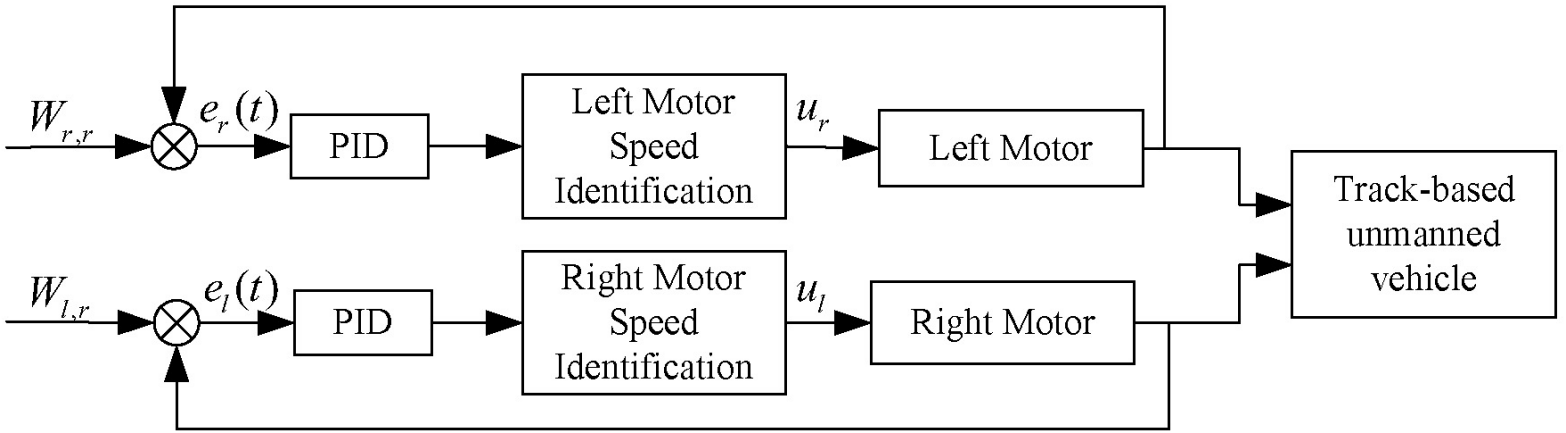
Motor speed controller.

By utilizing the previously identified Hammerstein-Wiener model of the current–hydraulic motor speed relationship, we developed a motor speed controller as shown in Fig 6. Additionally, a PID controller is incorporated to mitigate the effects of external factors such as terrain variations. The motor speed controller uses the speed error signal $e_{l}(t)$ and $e_{r}(t)$ as its input and employs PID control to adjust the electromagnetic valve current, thereby regulating the pump displacement to ensure that the actual vehicle speed aligns with the target speed.


$$\begin{array}{c} u(t)=K_{p}\cdot e(t)+K_{i}\int_{0}^{t}e(\tau )d\tau +K_{d}\frac{de(t)}{dt} \end{array}$$ (37)

where *e*(*t*) represents the system error, $K_{p}$ is the proportional gain, $K_{i}$ is the integral gain, and $K_{d}$ is the derivative gain.

## Experiment

### Experiment platform and experiment condition

The effectiveness of the proposed method was assessed via trajectory tracking experiments conducted on a fully hydraulic tracked unmanned vehicle. The test platform had a mass of 8.8 tons and overall dimensions of 4.5 m × 2.2 m, and it was equipped with an industrial PC (AIMB-T1215DA-00Y0E), a Huace 410 integrated navigation system, a PLC, and auxiliary devices. The software stack ran on Ubuntu 18.04 under the ROS Kinetic framework, enabling low-latency real-time data processing and reliable control.

Experiments were performed on a sandy soil field measuring 200 m × 50 m. Two reference trajectories were specified: a straight line and a circular curve with a radius of 15 m. The onboard IPC synchronously recorded vehicle speed, left and right hydraulic motor speeds, and pose information (latitude, longitude, heading angle) with a sampling period of 0.05 s. The experimental program consisted of speed tracking tests and trajectory tracking tests. Owing to limitations in test conditions, the speed tracking test, straight-line trajectory tracking test, and curved trajectory tracking test were each conducted once.

For comparative evaluation, a conventional MPC + PID scheme was selected as the experimental baseline. This scheme represents a mature engineering solution for trajectory tracking control of hydrostatic drive tracked unmanned vehicles, in which PID is the mainstream approach for low-level actuation control. In simulation comparisons, only traditional MPC was considered to directly quantify the performance gain introduced by the proposed MPC algorithm, whereas the complete MPC + PID architecture was implemented in the experiments to align with practical engineering applications. The baselines and the proposed method share consistent control objectives and application scenarios, thereby supporting objective and credible performance verification.

### Simulation experiments

Traditional MPC exhibits a fundamental trade-off in autonomous driving trajectory tracking. Improving tracking accuracy typically requires extending the prediction and control horizons ($N_{P}/N_{C}$); however, longer horizons substantially increase the number of decision variables in the control sequence Δ𝐔. As a result, the computational cost of quadratic programming (QP) solving grows rapidly, making it challenging to satisfy real-time requirements in autonomous driving. In contrast, reducing the horizon to enhance real-time performance weakens prediction capability and increases tracking error. To address this trade-off and evaluate the performance of Laguerre function-based MPC (Laguerre-MPC), simulation experiments are conducted in the MATLAB environment in this section, comparing the control performance of traditional MPC with the Laguerre-MPC developed in this paper.

**Fig 7:**
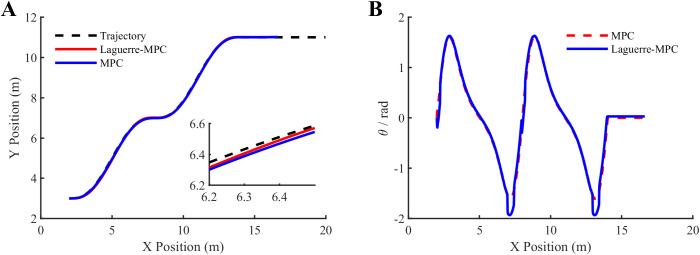
Laguerre-MPC and MPC simulation verification: (A) trajectory tracking; (B) vehicle heading angle.

Simulation studies were conducted in MATLAB to evaluate the performance of the Laguerre-based MPC strategy relative to a conventional MPC method. The trajectory tracking results in Fig 7A show higher tracking accuracy for Laguerre-MPC, as further evidenced by the magnified inset in the lower-right corner of Fig 7A. In contrast, the heading angle response in Fig 7B exhibits abrupt variations for Laguerre-MPC at the 7 m and 13 m positions. These discontinuities are attributed to rapid heading-error correction near trajectory inflection points, which induces sharp changes in control inputs and suggests increased sensitivity to trajectory deviations. Computational efficiency was further assessed using the MATLAB tic and toc functions. As reported in Table 1, Laguerre-MPC required 0.71 s, whereas conventional MPC required 2.94 s, corresponding to 24.3% of the conventional MPC runtime. This reduction highlights the computational advantage of the proposed strategy.

**Table 1:** Comparison of program running time.

Control methods	Solving time(s)
MPC	2.94
Laguerre-MPC	0.71

### Velocity tracking experiments

Velocity tracking experiments were carried out to validate the effectiveness of a system identification-based motor speed control technique, with a conventional PID controller serving as a baseline for comparison. Target speeds were set at 1.5 km/h and 3 km/h, with a sampling interval of 0.05s. Controller parameters are summarized in Table 2.

**Table 2:** Controller parameters for velocity tracking.

Parameters	Proposed Controller	PID
$K_{p}$	0.2	0.1
$K_{i}$	2.5	0.1
$K_{d}$	0.1	0.1

**Fig 8:**
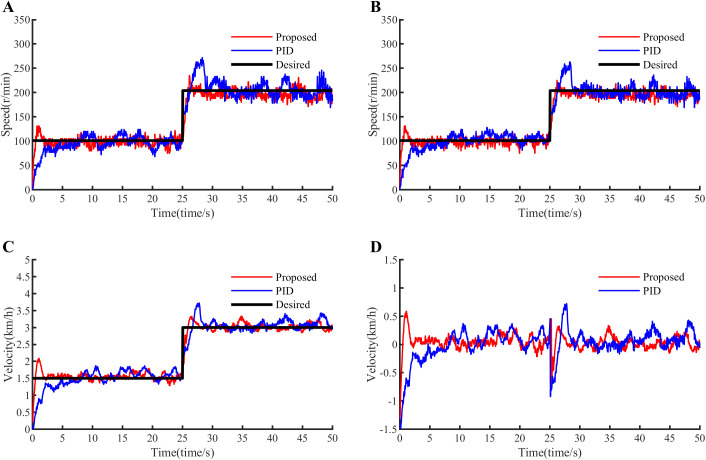
Speed tracking test: **(A)** Left motor speed; **(B)** Right motor speed; **(C)** Expected vehicle speed; **(D)** Expected vehicle speed error.

Results for the left and right hydraulic motors, depicted in Fig 8A and 8B, indicate that the developed technique yields lower tracking errors and better alignment with reference values compared to PID control. Furthermore, vehicle speed tracking data in Fig 8C and error profiles in Fig 8D reveal that while the PID controller exhibits notable overshoot, the proposed method stabilizes rapidly at the setpoint with minimal overshoot and a substantially shorter settling time.

### Trajectory tracking experiments

Trajectory tracking tests were conducted to verify the performance of the proposed hierarchical control architecture, encompassing both straight-line and curved path scenarios. In the straight-line trajectory tracking test, two coordinate points were collected using RTK as the starting and ending points, with a distance of 100 meters between them. The line connecting these two points served as the reference trajectory for the straight-line tracking test. In the curve tracking test, six coordinate points were collected and connected to form an S-shaped curve, with adjacent points spaced 50 meters apart and a total length of 200 meters, which served as the reference trajectory for the curve tracking test. A comparative control scheme, combining MPC as the upper-layer controller and PID as the lower-layer controller (denoted as MPC + PID), was also evaluated. The reference velocity was set to 1.5 km/h, with a sampling period of 0.05s, and relevant controller parameters are listed in Table 3.

**Table 3:** Parameters of controller.

Parameters	Proposed Controller	MPC + PID
$K_{p}$	0.2	0.2, 0.1
$K_{i}$	0.1	0.1
$K_{d}$	0.1	0.1
$N_{p}$	10	10
$N_{c}$	10	10
Weighting Matrix	*Q* = eye(3);*R* = diag([1.0695,1.0695])	*Q* = diag([10,10,1]);*R* = eye(2)
α	$\alpha_{1}=0.35;\alpha_{2}=0.55$	\
Parameters	$n_{1}=3;n_{2}=3$	\

**Fig 9:**
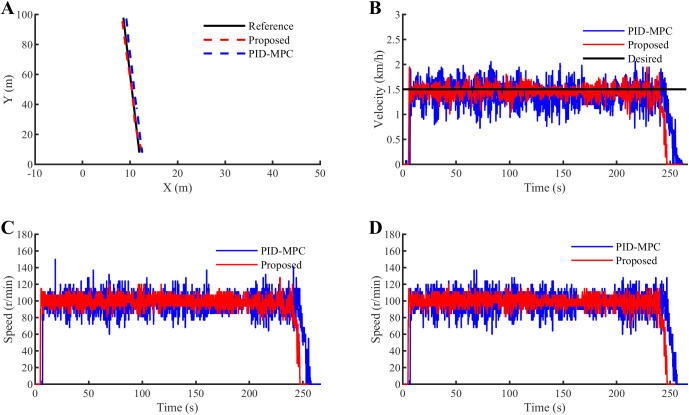
Tracking results of the linear trajectory: **(A)** Trajectory; **(B)** Vehicle speed; **(C)** Left-side velocity; **(D)** Right-side velocity.

For straight-line trajectories, results in Fig 9A demonstrate that the developed strategy achieves exceptional convergence to the reference path, surpassing the MPC + PID approach in both accuracy and stability. Velocity profiles in Fig 9B further show reduced fluctuations in actual speed compared to the baseline method. Moreover, rotational speed responses of the left and right hydraulic motors, presented in Fig 9C and 9D, highlight improved dynamic stability under coordinated dual-motor control with the proposed framework.

**Fig 10:**
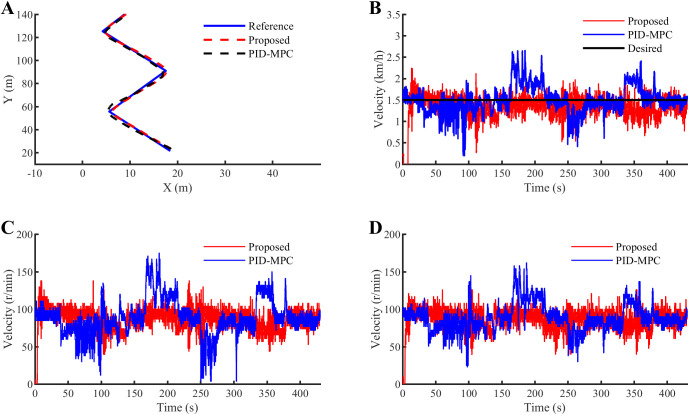
Tracking results of the curve trajectory: **(A)** Trajectory; **(B)** Vehicle speed; **(C)** Left-side velocity; **(D)** Right-side velocity.

In curved trajectory tests, Fig 10A indicates that the proposed strategy maintains higher tracking accuracy, particularly in the initial phase, consistently outperforming MPC + PID despite a gradual decline in precision over time for both methods. Comparative analysis of vehicle speed and motor speeds in Fig 10B-10D reveals significant overshoot and variability in MPC + PID during turns, reflecting limited disturbance rejection. In contrast, the outputs of the proposed method are notably smoother, demonstrating stronger robustness under challenging conditions.

**Fig 11:**
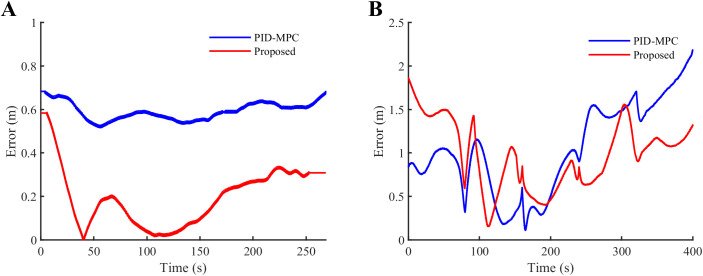
Tracking errors for straight-line and curved trajectories: **(A)** Tracking error of straight-line trajectory; **(B)** Tracking error of curved trajectory.

The results of the tracking error for the two experiments are depicted in Fig 11, with the maximum error, root mean square error (RMSE), and mean error (ME) calculated and summarized in Table 4. Since GPS was utilized to supply positioning data during the entire experimental process, the unmanned vehicle demonstrated inaccurate parking positions at the starting points of both the straight-line and curved trajectory tracking tests. As a result, the error calculation began after 40 seconds. The results indicate that the tracking performance for both straight-line and curved trajectories is superior to that of the conventional MPC + PID trajectory tracking control.

**Table 4:** Tracking errors of the trajectory tracking experiment.

Position Errors (m)		straight-line trajectory	curved trajectory
Proposed Controller	Max	0.34	1.8619
	ME	0.17	0.9775
	RMSE	0.20	1.0487
MPC + PID	Max	0.64	2.1894
	ME	0.58	1.0453
	RMSE	0.58	1.1667

Straight-Line trajectory scenario: Under the straight-line tracking condition, the proposed controller achieves substantially improved tracking accuracy. The maximum position error is 0.34 m, representing a 46.9% reduction relative to the 0.64 m obtained with the MPC + PID controller. The mean position error is 0.17 m, corresponding to a 70.7% decrease compared with the 0.58 m of the baseline method. The root mean square error is 0.20 m, indicating a 65.5% reduction relative to the 0.58 m of the baseline method. These results indicate that the proposed control method effectively compensates for hydraulic system disturbances, improves speed stability, and supports high-precision straight-line trajectory tracking.

Under the curved-trajectory scenario, the absolute errors of both controllers increased because vehicle steering introduced complex disturbances, including track slip and dynamic coupling between the left and right hydraulic motors. Despite these effects, the proposed controller consistently maintained a performance advantage: the maximum position error was 1.8619 m, representing a 14.9% reduction compared with 2.1894 m for the conventional scheme; the mean error was 0.9775 m, corresponding to a 6.5% decrease relative to 1.0453 m; and the RMSE was 1.0487 m, achieving a 10.1% reduction compared with 1.1667 m. The improved accuracy under curved conditions further confirms the robustness of the proposed hierarchical control architecture. During steering with constraints, Laguerre-MPC continues to generate accurate steering-angle and speed commands at low computational complexity. In addition, the identification model accurately captures the nonlinear characteristics of the hydraulic motors, thereby suppressing pressure fluctuations and rotational-speed deviations in the hydraulic system during steering and sustaining trajectory-tracking performance under complex operating conditions.

Vehicle experiments indicate that the proposed controller improves trajectory-tracking accuracy and demonstrates strong engineering applicability.

## Conclusions and discussion

To address the high computational complexity of MPC for hydraulically driven tracked unmanned vehicles and the difficulty of obtaining an accurate dynamic model of hydraulic drive systems using conventional approaches, a hierarchical control architecture is developed to enhance trajectory-tracking performance.

In the upper-layer design, Laguerre orthogonal basis function-based MPC (Laguerre-MPC) is applied to vehicle kinematic control. By exploiting the properties of Laguerre orthogonal basis functions, the control variables are effectively reduced in dimension, which substantially decreases the computational burden and mitigates the real-time limitations encountered when MPC is implemented on hydraulically driven tracked unmanned vehicles.

In the lower-layer design, to overcome the limitations of traditional physical-based modeling in representing hydraulic-motor nonlinearities, including high complexity, limited accuracy, and poor adaptability to practical engineering scenarios, a Hammerstein-Wiener model is adopted to describe the current-speed relationship of the hydraulic motors. PID control is further integrated to provide real-time disturbance compensation, ensuring reliable execution of the upper-layer trajectory-tracking commands.

Future work will further extend and refine the proposed control architecture: (1) for modeling the current-rotational speed characteristics of hydraulic motors, physics-informed neural networks will be introduced to achieve high-precision identification of hydraulic-system dynamics; (2) for data acquisition, the single unidirectional incremental excitation signal and bench tests will be replaced by data collection under typical operating conditions during real-vehicle ground driving; (3) for track slip and vehicle-terrain interaction dynamics, reinforcement learning will be employed to adaptively optimize the track-ground interaction model, improving anti-slip robustness in off-road environments; and (4) multi-sensor fusion integrating lidar and camera data will be incorporated to enable real-time environmental perception and autonomous navigation in complex dynamic scenarios. These developments are expected to further improve the autonomous control system of hydraulically driven tracked unmanned vehicles and facilitate engineering deployment.
